# Feasibility and Repeatability of Handheld Optical Coherence Tomography in Children With Craniosynostosis

**DOI:** 10.1167/tvst.10.8.24

**Published:** 2021-07-27

**Authors:** Sohaib R. Rufai, Richard Bowman, Catey Bunce, Vasiliki Panteli, Rebecca J. McLean, Seema Teli, Irene Gottlob, Mervyn G. Thomas, Noor ul Owase Jeelani, Frank A. Proudlock

**Affiliations:** 1 Clinical and Academic Department of Ophthalmology, Great Ormond Street Hospital for Children NHS Foundation Trust, London, UK; 2 UCL Great Ormond Street Institute of Child Health, London, UK; 3 The University of Leicester Ulverscroft Eye Unit, Leicester Royal Infirmary, Leicester, UK; 4 Craniofacial Unit, Great Ormond Street Hospital for Children NHS Foundation Trust, London, UK; 5 Clinical Trials Unit, The Royal Marsden NHS Foundation Trust, London, UK

**Keywords:** craniosynostosis, intracranial pressure, intracranial hypertension, papilledema, optical coherence tomography, feasibility, repeatability

## Abstract

**Purpose:**

To determine whether handheld optical coherence tomography (OCT) is feasible and repeatable in children with craniosynostosis.

**Methods:**

This was a prospective cross-sectional study. Children with syndromic and non-syndromic craniosynostosis 0 to 18 years of age were recruited between February 13, 2020, and October 1, 2020. Main outcome measures included feasibility (patient recruitment and handheld OCT success rates) and repeatability, which were assessed using intraclass correlation coefficients (ICCs) where repeated images of the optic nerve head (ONH) within the same visit were available. ONH parameters used for repeatability analysis included cup depth, width, and area; disc width; rim height; retinal thickness; retinal nerve fiber layer thickness; and Bruch's membrane opening minimum rim width.

**Results:**

Fifty children were approached, and all 50 (100%) were successfully recruited. Median age was 51.1 months (range, 1.9–156.9; interquartile range, 37.0–74.2), and 33 of the children (66%) were male. At least one ONH image was obtained in 43 children (86%), and bilateral ONH imaging was successful in 38 children (76%). Factors boosting the likelihood of success included good understanding and cooperation of the child and parent/guardian and availability of an assistant. Repeatability analysis was performed in 20 children, demonstrating good repeatability (ICC range, 0.77–0.99; the majority exceeded 0.90). OCT correctly identified two cases of intracranial hypertension, one of which was undetected by prior fundoscopy.

**Conclusions:**

Handheld OCT is feasible and repeatable in children with syndromic and non-syndromic forms of craniosynostosis.

**Translational Relevance:**

Our handheld OCT approach could be used for the clinical surveillance of children with craniosynostosis.

## Introduction

Craniosynostosis is characterized by the premature fusion of the cranial sutures, with an estimated prevalence of 3.1 to 6.4 per 10,000 births and rising.^1^ It is commonly associated with pathologically raised intracranial pressure (ICP), termed intracranial hypertension (IH). If unaddressed, IH can lead to visual impairment, cognitive impairment, and death. Craniosynostosis can be non-syndromic or syndromic.^2^ Among non-syndromic cases, IH typically occurs in 17%^3^ where a single suture is fused and in 24% to 47%^4^^,^^5^ where multiple sutures are fused. IH affects 30% to 40%^6^^,^^7^ of syndromic cases overall, most commonly including Apert^8^ (71%), Crouzon^9^ (61%), and Pfeiffer^5^ (60%) syndromes. Neurosurgical intervention involves expanding the skull vault to prevent the devastating sequelae of IH.

Recognizing IH in children is difficult. The gold-standard method is direct intracranial ICP measurement, but this requires hospital admission and carries risks relating to general anesthesia, infection, bleeding, leakage of cerebrospinal fluid, and mechanical failure; another limitation is difficulty in obtaining serial, repeated measures.^7^ An ideal measure of ICP should be non-invasive, highly sensitive, objective, and child friendly and should permit serial measurements to detect early changes associated with IH. The existing non-invasive measures that fail to fulfill all of these criteria include fundoscopic examination,^10^ transorbital ultrasound,^11^ and radiography,^12^ each delivering inadequate sensitivity to be used as effective surveillance tools.

**Figure 1.**
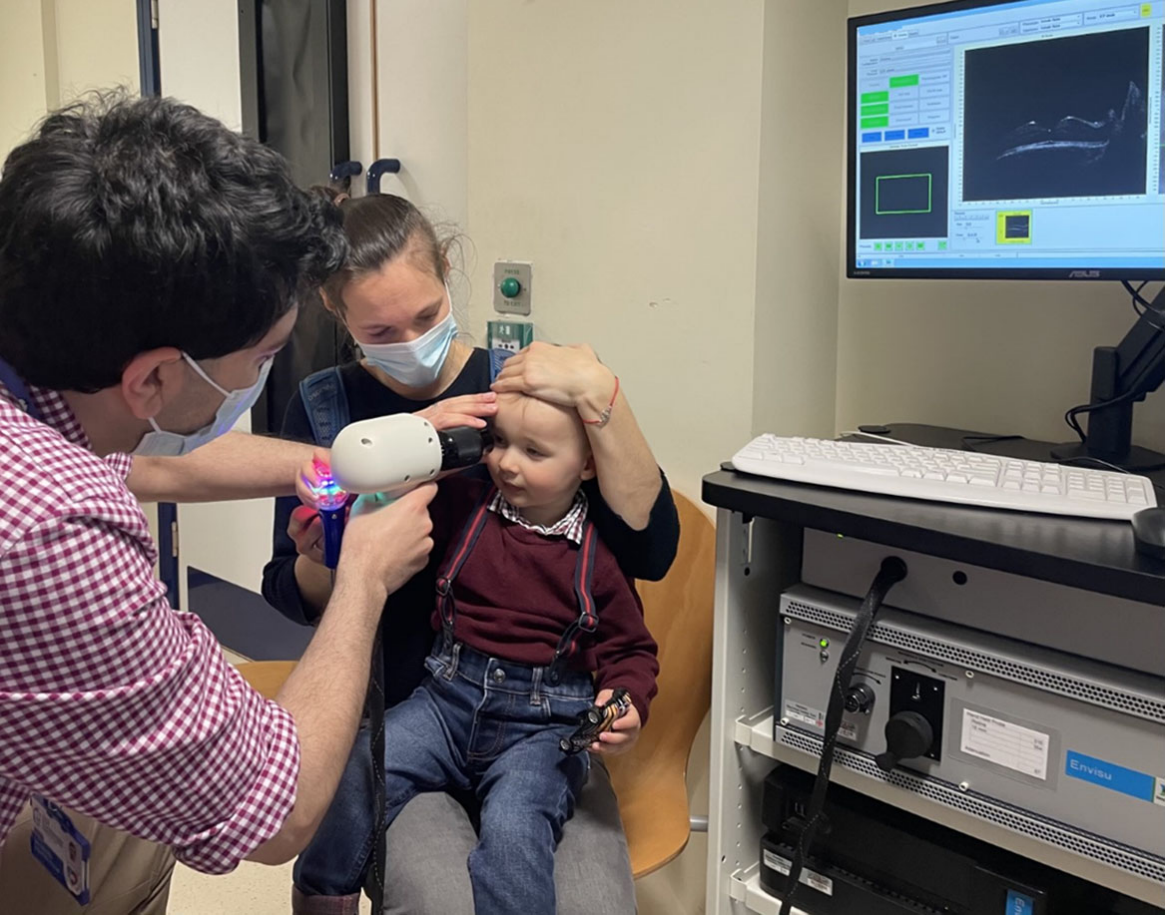
Handheld OCT examination in young child with craniosynostosis.

IH can cause swelling of the optic nerve, termed papilledema. If prolonged, this can lead to optic atrophy, causing irreversible vision loss. Optical coherence tomography (OCT) is a non-invasive, ultrahigh-resolution imaging modality that generates cross-sectional scans of the optic nerve and retina within seconds. A handheld OCT device has recently been developed for the pediatric population (Fig. 1). Although our craniofacial unit has utilized other ophthalmological investigations in children with craniosynostosis,^13^ we had not yet utilized handheld OCT in these patients prior to this study. Handheld OCT has been utilized to describe the normal development of the optic nerve^14^ and fovea^15^ in children, with excellent feasibility. Furthermore, handheld OCT has been utilized in numerous pediatric conditions,^16^ including primary congenital glaucoma,^17^ nystagmus,^18^ foveal hypoplasia,^19^^,^^20^ optic nerve hypoplasia,^21^ achromatopsia,^22^ retinopathy of prematurity,^23^ and craniopagus twins,^24^ among others. Other OCT devices have demonstrated potential for detecting papilledema and IH in craniosynostosis, but handheld OCT has not yet been used in this patient population.^25^ Although standard, table-mounted OCT devices may be used in cooperative children as young as 3 years of age, particularly with faster systems featuring eye-tracking capabilities, handheld OCT is feasible in infants from birth, without sedation, in the office setting.^14^^,^^25^ Handheld OCT is also designed to be used in the operating theater for patients under general anesthesia, as it is portable and wireless (battery-powered). Early recognition of papilledema during the first few years of life is crucial in this patient population; hence, handheld OCT may present unique advantages in infants and young children with craniosynostosis.

The primary objective of this study was to assess the feasibility of handheld OCT in children with craniosynostosis, defined as recruitment success rate and image acquisition success rate. The secondary objective was to perform repeatability analysis of our ONH parameters.

## Methods

### Study Design and Participants

This was a prospective cross-sectional study conducted at Great Ormond Street Hospital for Children (GOSH). This study adhered to the tenets of the Declaration of Helsinki. Ethical approval was granted by the East Midlands Nottingham 2 Research Ethics Committee (UOL0348/IRAS 105137). This study was reported as per the Strengthening the Reporting of Observational Studies in Epidemiology (STROBE) statement.^26^

All patients presenting to the GOSH craniofacial all-day clinic on Thursdays between February 13, 2020, and October 1, 2020, were approached in the eye department for recruitment. In addition, patients listed for ICP assessment were approached in the surgical admissions ward for recruitment. All patients were approached regardless of age or suspicion surrounding cooperation. The recruitment period was predetermined to ensure sufficient recruitment without delaying our larger prospective study: Recognition of Intracranial Hypertension in Children Using Handheld OCT (The RIO Study; ISRCTN52858719). Inclusion criteria were as follows: (1) diagnosis of craniosynostosis, (2) age range 0 to 18 years, and (3) parent/guardian available to provide consent. Children without an available parent/guardian to provide consent were excluded.

### Outcome Measures

Main outcome measures for feasibility included patient recruitment success rate and handheld OCT image acquisition success rate, described at the following levels: (1) At least one optic nerve head (ONH) image acquired in either eye of analyzable quality; and (2) bilateral ONH images acquired of analyzable quality. Analyzable quality was defined as an ONH tomogram wherein the edges of the disc margins and the cup profile, including its lowermost point, were clearly visualized. The scan was repeated twice per eye where possible to permit repeatability analysis. If unsuccessful, scans were repeated until images of satisfactory quality were achieved or until the child ceased to take part. Total research clinic time was up to 10 minutes per participant.

Other factors associated with feasibility were recorded as follows:

• Mode of examination (i.e., research clinic without sedation or under general anesthesia in the operating theater)
• Availability of an assistant during research clinic examinations
• Reasons for non-acquisition

All handheld OCT examinations were performed by the lead investigator (SRR). If an assistant was available, that person would operate the computer to acquire images, as well as help to keep the child engaged using visual fixation devices. Wherever an assistant was not available, the lead investigator performed the OCT examination alone using a foot pedal. No patients were intentionally dilated for the purposes of this study.

The secondary outcome measure was the repeatability of OCT parameters of the ONH within the same visit where available. The following background information was collected: diagnosis (clinical and/or genetic), sex, age at examination, fundoscopic examination findings (performed by consultant neuroophthalmologist), visual acuity, and 48-hour ICP assessment outcomes (ICP bolt; Raumedic AG, Helmbrechts, Germany), where available.

### Handheld OCT Image Acquisition and Analysis

A non-contact, spectral-domain, handheld OCT device (Envisu C-2300 with 3.3-µm axial resolution and 11-µm lateral resolution; Leica Microsystems, Wetzlar, Germany) was used in this study. Examinations were performed in children in an outpatient setting without sedation and, in some cases, under general anesthesia immediately prior to ICP bolt insertion or on the surgical ward. Visual fixation devices, including cartoons on smartphones or tablets and toys, were used to minimize movement during image acquisition in awake children. The acquisition protocol used a 12 × 8-mm scanning window and short acquisition time (1.9 seconds) to facilitate successful acquisition with minimal disruption of image quality, to avoid measurement bias. The three-dimensional raster scan program for both scan sequences was comprised of 100 B-scans and 500 A-scans per B-scan line.

**Figure 2.**
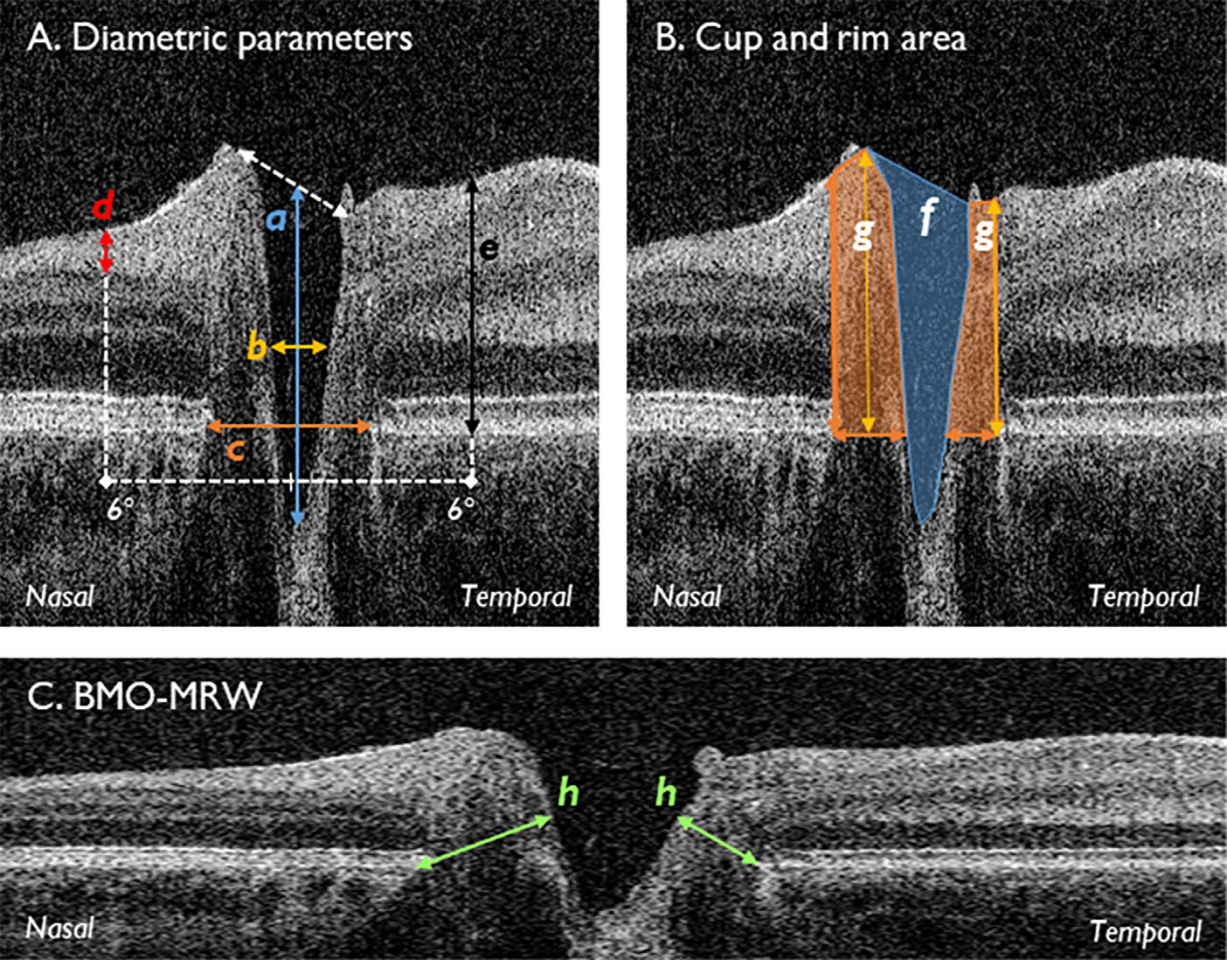
Segmentation parameters. (A) Diametric parameters: (a) cup depth (*blue*), measured from cup base to midpoint of neuroretinal peaks; (b) cup diameter (*amber*), measured at midpoint of cup depth; (c) disc diameter (*orange*), measured from nasal to temporal Bruch's membrane; (d) RNFL thickness (*red*), measured at 6° from the disc midpoint, bounded by the ILM and GCL; (e) retinal thickness (*black*) measured at 6° from the disc midpoint, bounded by the ILM and Bruch's membrane. (B) Cup and rim area: (f) cup area (*blue shade*), bounded by neuroretinal peaks; (g) rim area (*orange shade*), bounded by edges of Bruch's membrane; rim width, represented by lower-most borders of (g); rim height, maximum distance between rim width and ILM, represented by amber arrows within (g). (C) Natural scale image displaying the BMO-MRW (h). Nasal and temporal measurements were taken for (d), (e), (g), and (h), plus rim height. BMO-MRW, Bruch's membrane opening minimum rim width; GCL, ganglion cell layer; ILM, internal limiting membrane; RNFL, retinal nerve fiber layer thickness.

Our handheld OCT imaging protocol involved scanning the right eye followed by the left eye. The en face view was used to identify the ONH, which was navigated frame by frame until the central slice featuring the deepest optic cup was identified for ONH analysis. Scans were repeated twice per eye where possible to permit repeatability analysis, minimized to the least time possible between repeated scans and within the 10-minute imaging session to avoid potential confounders. ImageJ 1.48 (National Institutes of Health, Bethesda, MD)^27^ was used for quantitative segmentation to permit repeatability analysis; the ABSnake plugin^28^ was used to identify the internal limiting membrane contour, which was corrected manually where required. All quantitative segmentation analysis was performed by the lead investigator (SRR). The lateral distance measurements (defined for adults on the machine) were corrected to account for the smaller axial lengths in the infant population using a conversion table according to age from the data presented by Maldonado et al.^29^ Figure 2 displays the segmentation parameters used for the repeatability analysis. Interexaminer reliability between two independent graders using the same analysis method has previously been demonstrated to be high in 30 children.^14^

### Statistical Analysis

Statistical analysis was performed using SPSS Statistics 22.0. (IBM Corp., Armonk, NY). Demographic data were summarized using descriptive statistics. The recruitment success rate was reported as a percentage, and the unilateral and bilateral ONH image acquisition success rates were reported as percentages.

Repeatability analysis was performed as follows. Quantitative segmentation was performed for Image 1 and Image 2, wherever two images were available from the same eye within the same visit. Overall mean figures for the included OCT parameters were calculated and determined to be approximately normally distributed. Mean differences for the included OCT parameters between Image 1 and Image 2 were calculated. Intraclass correlation coefficients (ICCs) were calculated for all ONH parameters using single measures in a two-way mixed-effects model.^30^ Finally, 95% limits of agreement were calculated as mean difference ± 1.96 SD of the difference.

## Results

### Baseline Demographics

Out of 50 children approached for recruitment, 50 (100%) were successfully recruited. Of all 50 included children, median age was 51.1 months (range, 1.9–156.9; interquartile range [IQR], 37.0–74.2); 33 of the children (66%) were male; and 13 of the children (26%) had syndromic craniosynostosis.

### Feasibility

**Figure 3.**
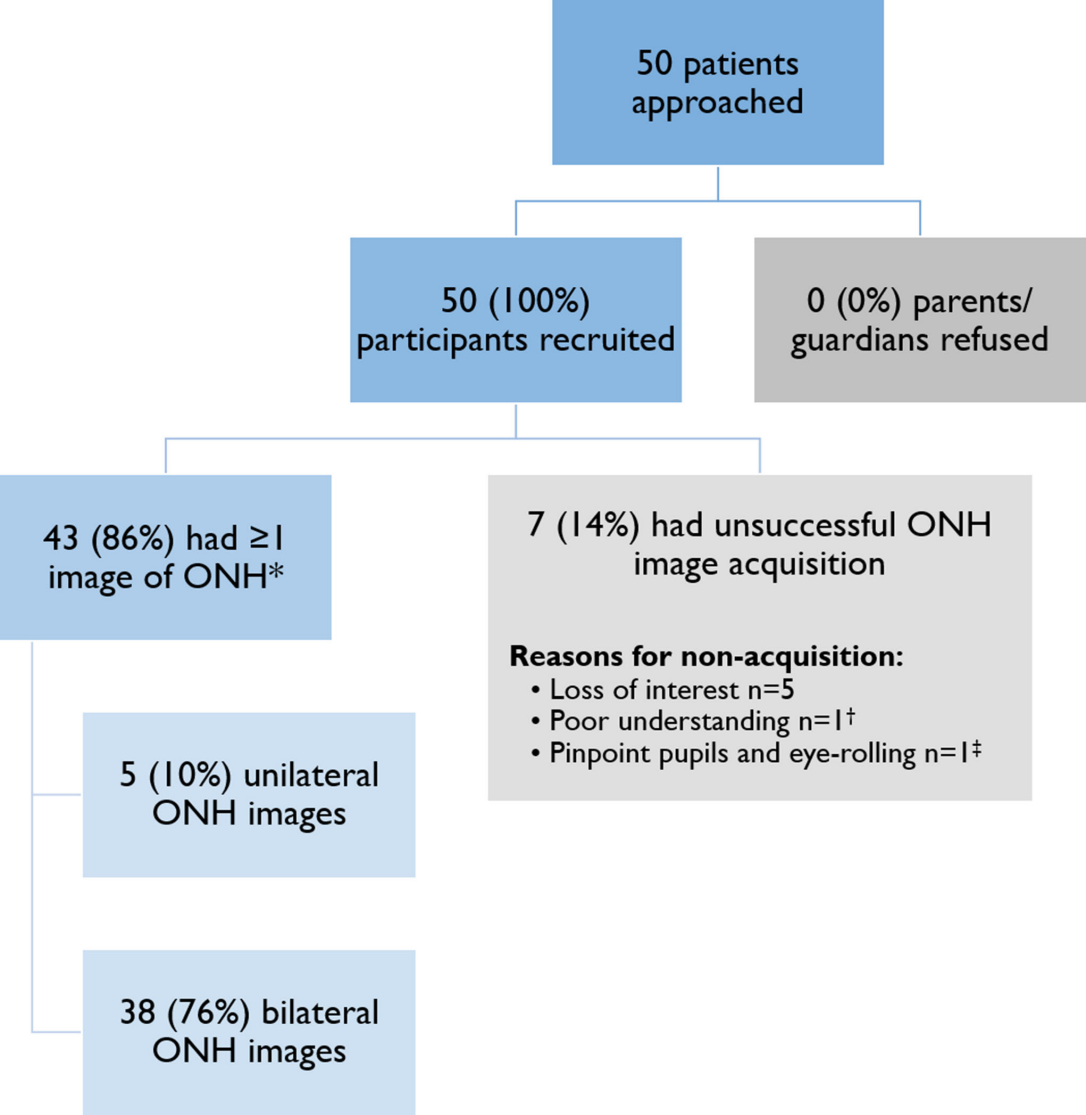
Feasibility flowchart. ^*^When excluding four children imaged under general anesthesia, 39 out of 46 of the children (84.8%) achieved ≥1 ONH image, and 35 out of the 46 children (76.1%) achieved bilateral ONH images. ^†^Child with Crouzon syndrome and cognitive impairment had limited cooperation due to poor understanding, whereas all other unsuccessful attempts were in children with non-syndromic craniosynostosis. ^‡^Pinpoint pupils and eye-rolling were caused by opiate administration prior to handheld OCT examination in the operating theater.

Figure 3 displays the feasibility flowchart for this study. At least one ONH image of analyzable quality was successfully obtained in 43 children (86%), and bilateral ONH images were obtained in 38 children (76%). In the successful group (*n* = 43), the median age was slightly higher but the IQR was similar (median age, 66.3 months; IQR, 37.8–44.9; range, 1.9–156.9), as compared with the unsuccessful group (*n* = 7; median age, 37.8 months; IQR, 29.3–43.0; range, 20.1–70.9) (Table 1). In the successful group, 26 of the 43 children (60.5%) were male, whereas all seven children (100%) in the unsuccessful group were male. Twelve of the 43 children in the successful group had syndromic craniosynostosis (27.9%), compared with one of seven children in the unsuccessful group (14.3%). Bilateral ONH scans were successfully obtained in 38 children at first examination (76%).

**Table 1.** Patient Demographics

Baseline Characteristics	Patients With at Least One Successful ONH Scan	Patients With Unsuccessful ONH Scans
Diagnosis, *n*		
Syndromic		
Apert	4	—
Crouzon	3	1
Pfeiffer	2	—
Muenke	1	—
Bartter (type 4)	1	—
MEK2 mutation	1	—
Syndromic total	12	1
Non-syndromic		
Sagittal	20	5
Multisuture	11	1
Non-syndromic total	31	6
Gender, *n*		
Male	26	7
Female	17	0
Age at handheld OCT (mo), median (IQR; range)	66.3 (37.8–44.9; 1.9–156.9)	37.8 (29.3–43.0; 20.1–70.9)

After excluding four children who were imaged under general anesthesia, success rates remained almost identical (≥1 analyzable ONH image, 39 out of 46 successful [84.8%]; bilateral analyzable ONH images, 35 out of 46 successful [76.1%]). Supplementary Table S1 displays success rates per age group after excluding the four children imaged under general anesthesia. At least one ONH image was successful in ≥75% of all age groups, except toddlers 2 to 3 years of age (60%).

Of the 50 recruited patients, 45 were imaged without sedation in the research clinic, four were imaged under general anesthesia in the operating theater immediately prior to their ICP bolt insertion, and one was imaged while awake on the surgical ward with the ICP bolt in situ, 1 day after insertion. The latter occurred simply due to logistical issues. The youngest two participants recruited in the research clinic were babies (1.9 and 2.6 months old), and bilateral ONH images were successfully obtained in both while they were napping. All other children imaged in the research clinic were awake. At first examination, an assistant was available to operate the computer in all five patients imaged under general anesthesia (100%) and in 17 of 45 patients imaged in the research clinic without sedation (37.8%). All other examinations were performed by the lead investigator (SRR) without assistance, using a foot pedal. Forty patients were undilated during the handheld OCT examination. Ten patients were already dilated by a clinician during their clinical appointment prior to the handheld OCT examination, but none of the patients imaged under general anesthesia were dilated, as this would interfere with the postoperative neurological observation protocol on the recovery ward, which includes regular assessment of pupillary light reactions.

### Reasons for Non-Acquisition

No analyzable ONH image was acquired in seven patients, who were between 20.1 and 70.9 months of age (median age, 37.8 months). Reasons for non-acquisition in the research clinic included the child becoming restless and losing interest (*n* = 5 children with non-syndromic craniosynostosis) and limited cooperation due to poor understanding (*n* = 1 child with Crouzon syndrome and cognitive impairment). No assistant was available for five of the six clinic patients for whom ONH image acquisition was unsuccessful. No child appeared to be distressed by the handheld OCT device. Of the children examined under general anesthesia, the first had sagittal synostosis and the image acquisition was unsuccessful due to pinpoint pupils and upward eye-rolling, which was secondary to opiate administration by the anesthetist. Thereafter, we changed the order such that the handheld OCT examination took place prior to opiate administration, successfully avoiding this problem in all subsequent OCT acquisitions in the operating theater.

### Repeatability

**Figure 4.**
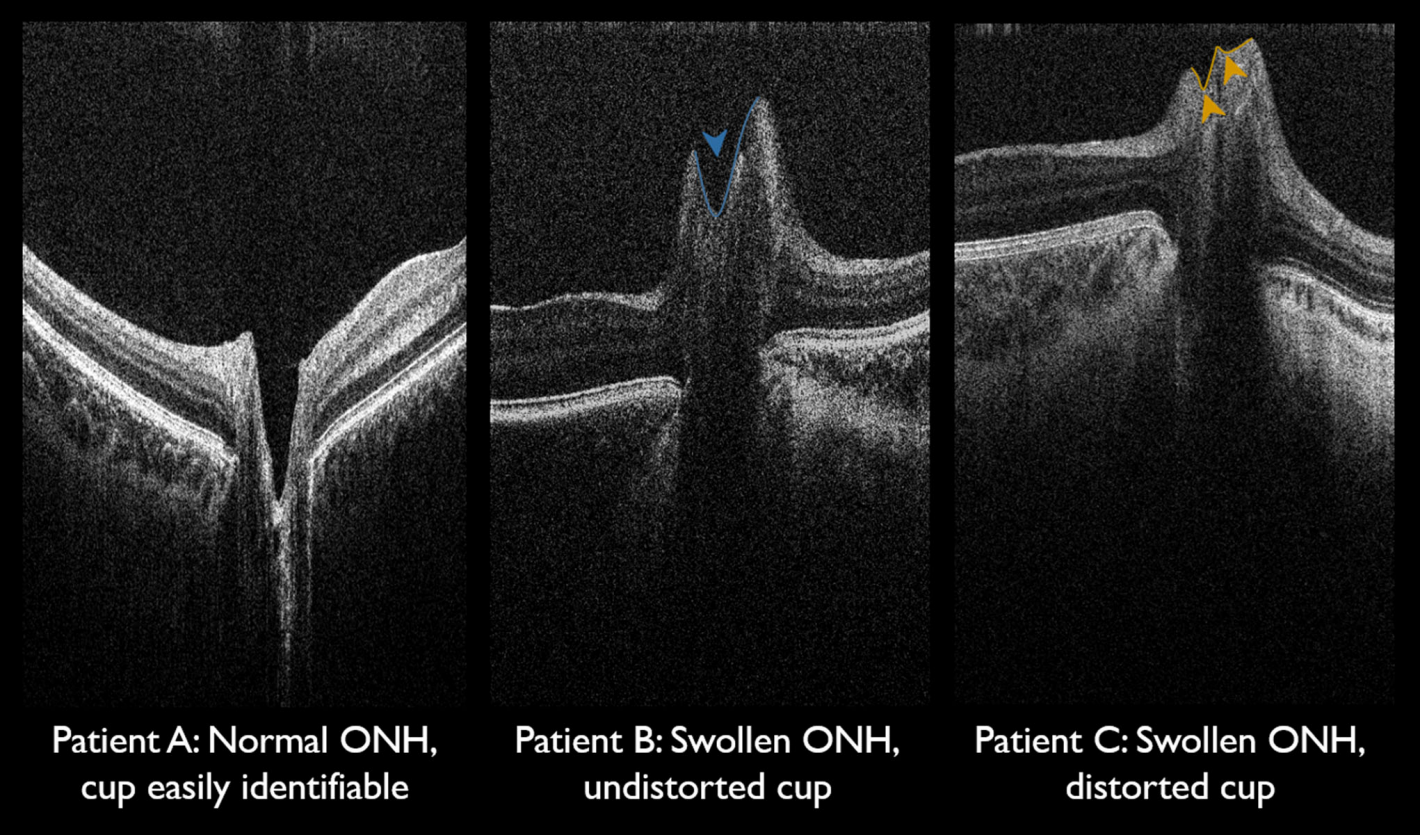
Variation in optic cup morphology. Patient A has a normal ONH with an easily identifiable cup in the form of a parabolic curve. Patient B has a swollen ONH, but the cup is still identifiable as a parabolic curve (*blue arrowhead* and *delineation*). Patient C has a swollen ONH, but the cup is more difficult to identify and landmark as the swelling has interrupted the typical parabolic curve (*gold arrowheads* and *delineation*). All three patients were included in the repeatability analysis. Patients B and C had intracranial hypertension proven on invasive intracranial pressure monitoring.

Repeatability analysis was possible using 54 ONH images available in 20 children out of 50 (40%), where repeated ONH images were available from the same eye during the same visit (Table 2). Repeatability analysis (Table 2) revealed good repeatability in all ONH parameters, with acceptable 95% limits of agreement. Temporal and nasal retinal nerve fiber layers (RNFLs) demonstrated ICCs of 0.77 and 0.81, respectively. All other ONH parameters demonstrated ICCs above 0.82, with the majority exceeding 0.90. During segmentation, the identification of cup landmarks required extra attention in shallow cups or swollen discs (Fig. 4). The optic cup normally resembles a parabolic curve. It was particularly challenging to segment the optic cup in one patient (Fig. 4, patient C) due to papilledema rendering the cup small and distorted, thus disrupting the usual parabolic curve, although the ICCs remained high for all cup parameters.

**Table 2.** Repeatability Analysis

OCT Parameters	Overall Mean	Mean Difference	ICC (95% CI)	95% Limits of Agreement
Optic nerve parameters				
Cup depth (µm)	326.67	1.35	0.99 (0.98–1.00)	−39.32, 42.02
Cup width (µm)	505.49	−23.16	0.82 (0.66–0.92)	−218.32, 172.00
Cup area (µm^2^)	171480.48	−1578.27	0.97 (0.93–0.99)	−52644.64, 49488.10
Disc width (µm)	1494.35	−9.38	0.91 (0.81–0.96)	−183.49, 164.73
Rim parameters				
Nasal rim height (µm)	467.87	1.73	0.99 (0.97–0.99)	−34.37, 37.83
Temporal rim height (µm)	314.45	2.39	0.99 (0.97–0.99)	−28.99, 33.77
Rim width (µm)	1354.30	−4.52	0.96 (0.91–0.98)	−180.12, 171.08
Rim area (µm^2^)	444491.31	−2900.64	0.99 (0.98–1.00)	−60257.75, 54456.47
Nasal retinal thickness (µm)	283.82	−1.27	0.87 (0.68–0.95)	−32.02, 29.48
Temporal retinal thickness (µm)	295.84	−0.55	0.94 (0.87–0.97)	−13.27, 12.17
Nasal RNFL thickness (µm)	70.22	1.86	0.81 (0.56–0.92)	−37.67, 41.39
Temporal RNFL thickness (µm)	57.61	1.22	0.77 (0.55–0.89)	−20.77, 23.21
Nasal BMO (µm)	417.20	−3.23	0.96 (0.91–0.98)	−67.38, 60.92
Temporal BMO (µm)	295.24	1.87	0.97 (0.94–0.99)	−32.61, 36.35

Fifty-four images from 20 children were included; mean difference in OCT parameters was derived from Image 1 versus Image 2, where both images were taken in the same eye on the same visit. The ICCs were obtained using a single-measure, two-way mixed effects model. CI, confidence interval; BMO, Bruch's membrane opening.

### Visual Acuity and Fundoscopy

Background information for visual acuity and fundoscopy in this cohort are summarized in Supplementary Table S2. Interestingly, in the patient with unsuccessful fundoscopy due to corneal scarring, handheld OCT was successfully achieved by tilting the handheld OCT probe around the corneal scar to visualize the ONH (Supplementary Figure S1). Papilledema was detected on same-day fundoscopy in patient C (Fig. 4), noted as modified Frisén grade 1.^31^ However, same-day fundoscopy in patient B (Fig. 4) demonstrated prominent discs but no clear papilledema. Optic atrophy was diagnosed on fundoscopy in one patient with syndromic craniosynostosis; RNFL thinning was also appreciated on handheld OCT performed on the same day, albeit image acquisition was only successful in the right eye due to limited patient understanding and cooperation.

### Intracranial Pressure Assessments

Forty-eight-hour ICP bolt assessments were available in five patients at the time of handheld OCT examination. Two ICP assessments excluded IH; both fundoscopic and handheld OCT findings were unremarkable. One ICP assessment demonstrated reduced intracranial compliance (normal ICP during daytime but raised overnight), but both fundoscopic and handheld OCT findings were unremarkable. Two ICP assessments demonstrated IH, wherein papilledema was present bilaterally on handheld OCT.

## Discussion

### Main Findings

This prospective cross-sectional study has demonstrated that handheld OCT is feasible in children with syndromic and non-syndromic forms of craniosynostosis. All 50 children and/or parents/guardians agreed to participate. The majority of handheld OCT examinations were successful, both in undilated/dilated non-anesthetized children in the research clinic and in undilated children under anesthesia in the operating theater. When analyzing success rates by age group (Supplementary Table S1), at least one ONH image was successful in ≥75% of all age groups, except toddlers 2 to 3 years of age (60%). This may be because externalizing behaviors and non-compliance are generally more common in toddlerhood but then improve in the preschool years.^32^

The majority of parents/guardians understood the test well and were committed to acquiring handheld OCT images; some successfully used the child's preferred cartoons on smartphones and/or toys as visual fixation devices. One child with Crouzon syndrome and associated cognitive impairment did not understand the test and hence could not cooperate. The main reason for non-acquisition was the participants becoming restless and losing interest; indeed, many had multiple prior clinical assessments and investigations, which could take several hours and involve numerous hospital departments. Handheld OCT image acquisition was feasible using the foot pedal, but images were easier to acquire with the help of an assistant, not only to acquire images by operating the computer but also to help keep the child engaged with visual fixation devices.

Good repeatability was demonstrated for all ONH parameters in 20 children. A small degree of variation in RNFL thickness may have occurred due to inadvertent tilting of the handheld OCT probe.^33^

### Research in Context

A large study by Patel et al.^14^ evaluated the feasibility of handheld OCT in healthy infants and children using the same Envisu C2300 device. Of 352 children ranging in age from 1 day to 13 years, the ONH of one eye was successfully imaged in 70% of participants. Using the same definition, our study demonstrated a higher handheld OCT success rate of 86% in children with craniosynostosis. This figure may be slightly higher due to the smaller sample size or the fact that the patients are more familiar with clinical investigations within a hospital environment. However, a substantial proportion of children with craniosynostosis, particularly syndromic craniosynostosis, have a degree of developmental delay.^34^ This presents challenges for handheld OCT examination, which we found can be overcome with the success factors listed above. Out of all ONH parameters, Patel et al.^14^ found that nasal and temporal RNFLs demonstrated the lowest interexaminer ICCs (0.77 and 0.68, respectively). Patel et al.^13^ also found that success rates dipped in toddlerhood and then increased with age, consistent with our findings.

Tran-Viet et al.^35^ recently found good feasibility for undilated handheld OCT examination using the Envisu C2300 in preterm infants and children with neurologic abnormalities; however, their study did not feature any children with craniosynostosis. Dilation may make it easier to identify the optic nerve or fovea when acquiring handheld OCT images. However, in our experience, dilating young children can sometimes make the patient distressed and less likely to cooperate; therefore, we do not routinely dilate specifically for handheld OCT examination.

Our recent systematic review^25^ identified only three studies using OCT in children with craniosynostosis. Swanson et al.^36^ used the iVue OCT device (Optovue, Fremont, CA; software version 3.2) in children under anesthesia and demonstrated good sensitivity and specificity for raised ICP using single ICP measures intraoperatively; OCT parameters included maximal RNFL thickness, maximal retinal thickness, and maximal anterior retinal projection. Our study demonstrated that handheld OCT could successfully be performed in infants as young as 1.9 months without general anesthesia. The median age of the 50 children in our study was 51.1 months; therefore, a significant proportion may potentially cooperate with table-mounted OCT. However, developmental delay is common in this patient population; although we did not specifically measure contemporaneous success rates with table-mounted OCT, we often find it is challenging even for older children with craniosynostosis. Swanson et al.^36^ also found high OCT intragrader reliability in 20 children, consistent with our findings.

### Limitations

This study has the limitations expected of a feasibility study. Our larger, ongoing prospective study (The RIO Study) aims to explore the role of OCT in recognizing IH in children. Recruitment was significantly impacted by the COVID-19 pandemic, as we were not permitted to recruit any participants from March 2020 to September 2020. Moreover, the number of patients per clinic was slightly reduced from September 2020 onward due to COVID-19 restrictions. Our sample of 20 patients for repeatability analysis was small, so caution should be exercised in interpreting or generalizing these findings. A larger sample size may have permitted more robust repeatability analysis and in-depth subgroup statistical analysis by age and other demographics. Four patients were imaged under general anesthesia, which removes the need for patient cooperation, albeit the success rates remained almost identical even when these four patients were hypothetically excluded, using the same definition of success.

## Conclusions

This study has demonstrated that handheld OCT is feasible and repeatable in children with syndromic and non-syndromic craniosynostosis. Further prospective research is required to determine whether handheld OCT represents a suitable surveillance tool for IH in children with craniosynostosis.

## Supplementary Material

Supplement 1

Supplement 2
