# Erratum in: Feasibility and Repeatability of Handheld Optical Coherence Tomography in Children With Craniosynostosis

**DOI:** 10.1167/tvst.12.12.16

**Published:** 2023-12-19

***Erratum in:*** “Feasibility and Repeatability of Handheld Optical Coherence Tomography in Children With Craniosynostosis,” by Sohaib R. Rufai, Richard Bowman, Catey Bunce, Vasiliki Panteli, Rebecca J. McLean, Seema Teli, Irene Gottlob, Mervyn G. Thomas, Noor ul Owase Jeelani, and Frank A. Proudlock (*Transl Vis Sci Technol.* 2021;10(8):24), https://doi.org/10.1167/tvst.10.8.24.

Figure 2 has been corrected in the article online. The orange shading representing the rim area (g) extends posteriorly to the level of Bruch's membrane in the corrected figure. The corrected legend includes two important clarifications: rim width is represented by the lower-most borders of (g) and rim height is the maximum distance between rim width and internal limiting membrane, represented by amber arrows within (g).

**Figure 2.**
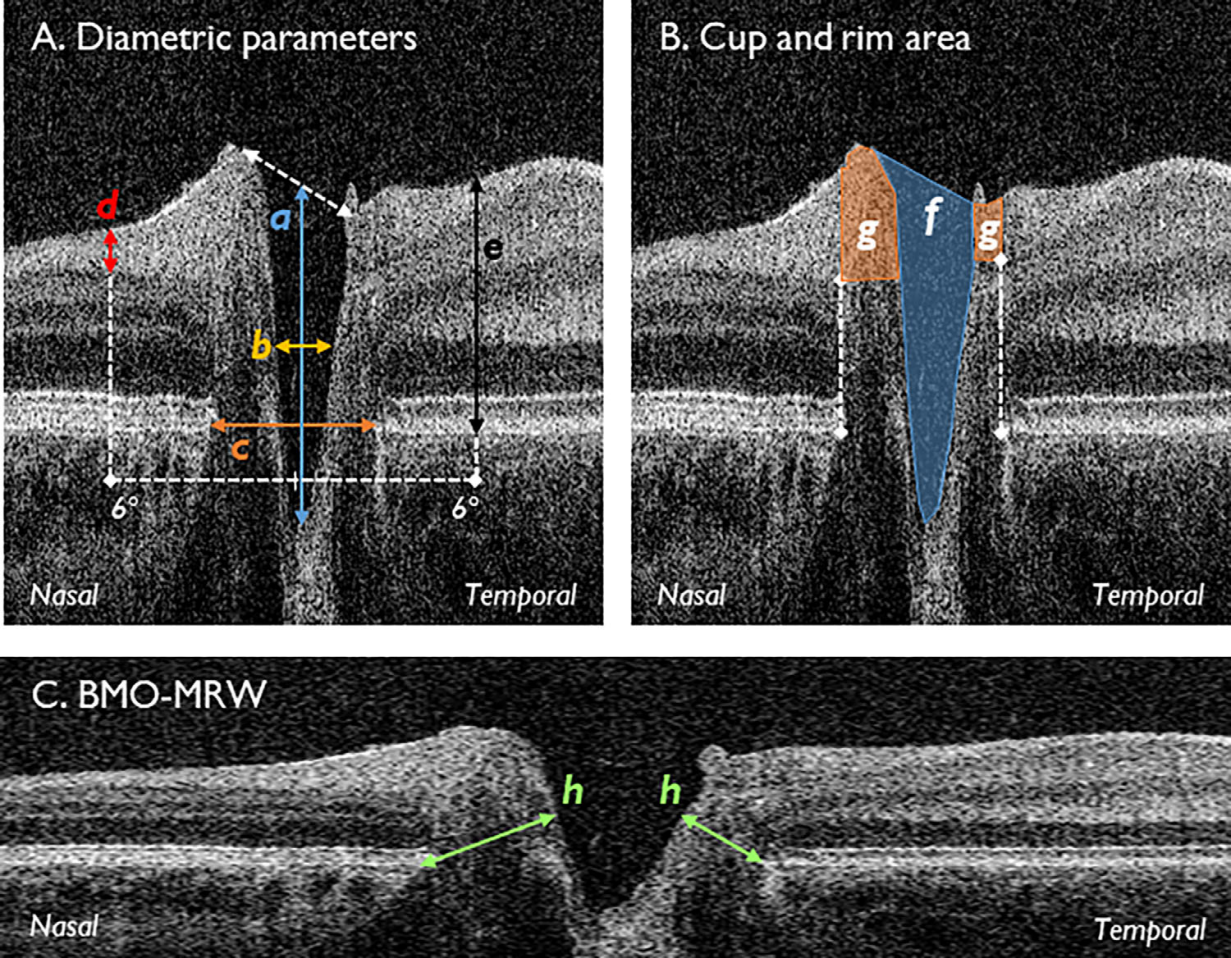
Original Figure 2

**Figure 2.**
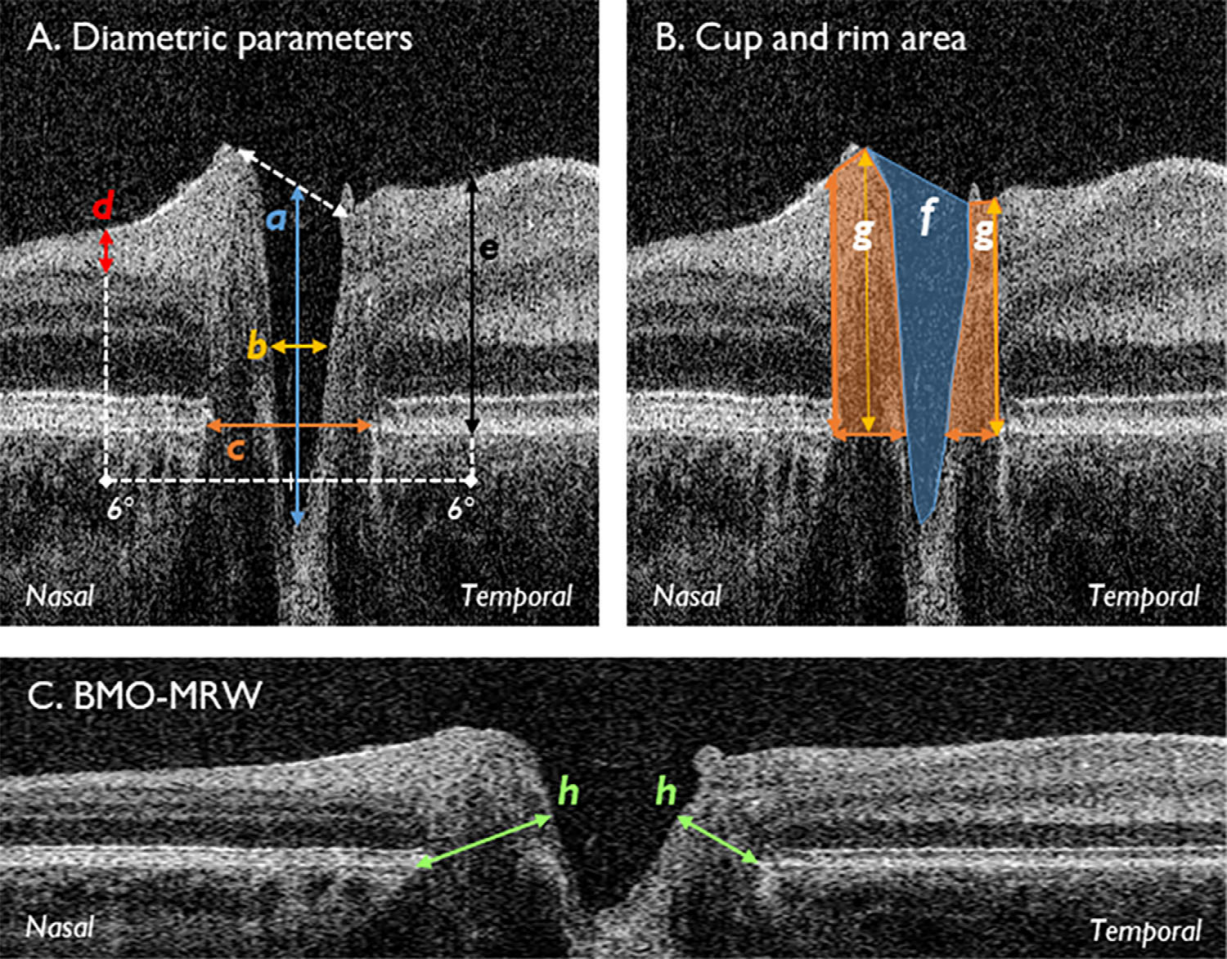
Corrected Figure 2

Original Figure 2 legend:

Segmentation parameters. (A) Diametric parameters: (a) cup depth (*blue*), measured from cup base to midpoint of neuroretinal peaks; (b) cup diameter (*amber*), measured at midpoint of cup depth; (c) disc diameter (*orange*), measured from nasal to temporal Bruch's membrane; (d) RNFL thickness (*red*), measured at 6° from the disc midpoint, bounded by the ILM and GCL; (e) retinal thickness (*black*) measured at 6° from the disc midpoint, bounded by the ILM and Bruch's membrane. (B) Cup and rim area: (f) cup area (*blue shade*), bounded by neuroretinal peaks; (g) rim area (*orange shade*), bounded by edges of Bruch's membrane. (C) Natural scale image displaying the BMO-MRW (h). Nasal and temporal measurements were taken for (d), (e), (g), and (h), plus rim height shown as the lateral borders of (g). BMO-MRW, Bruch's membrane opening minimum rim width; GCL, ganglion cell layer; ILM, internal limiting membrane; RNFL, retinal nerve fiber layer thickness.

Corrected Figure 2 legend (new text in bold):

Segmentation parameters. (A) Diametric parameters: (a) cup depth (*blue*), measured from cup base to midpoint of neuroretinal peaks; (b) cup diameter (*amber*), measured at midpoint of cup depth; (c) disc diameter (*orange*), measured from nasal to temporal Bruch's membrane; (d) RNFL thickness (*red*), measured at 6° from the disc midpoint, bounded by the ILM and GCL; (e) retinal thickness (*black*) measured at 6° from the disc midpoint, bounded by the ILM and Bruch's membrane. (B) Cup and rim area: (f) cup area (*blue shade*), bounded by neuroretinal peaks; (g) rim area (*orange shade*), bounded by edges of Bruch's membrane; **rim width, represented by lower-most borders of (g); rim height, maximum distance between rim width and ILM, represented by amber arrows within (g)**. (C) Natural scale image displaying the BMO-MRW (h). Nasal and temporal measurements were taken for (d), (e), (g), and (h), plus rim height. BMO-MRW, Bruch's membrane opening minimum rim width; GCL, ganglion cell layer; ILM, internal limiting membrane; RNFL, retinal nerve fiber layer thickness.

