# Supplementary material for: Feasibility and Repeatability of Handheld Optical Coherence Tomography in Children With Craniosynostosis
Source: Transl Vis Sci Technol. 2021 Jul 27;10(8):24. doi: 10.1167/tvst.10.8.24 (PMC8322722; doi:10.1167/tvst.10.8.24)

Supplementary Figure S1: Successful ONH scan in patient with left corneal scar. This was achieved by tilting the handheld OCT probe around the corneal scar to visualise the ONH. OCT = optical coherence tomography; ONH = optic nerve head.

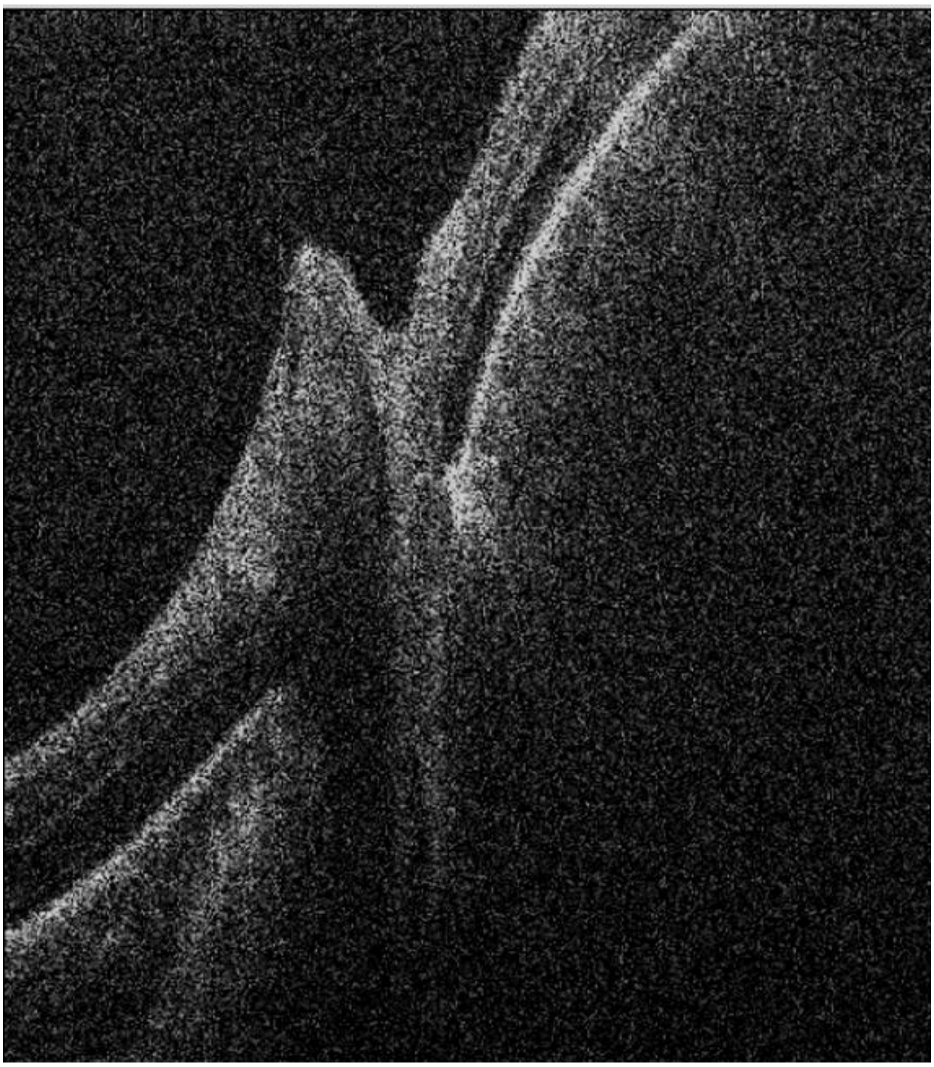

Supplement: Supplement 1 [file tvst-10-8-24_s001.pdf]
