# Supplementary material for: Feasibility and Repeatability of Handheld Optical Coherence Tomography in Children With Craniosynostosis
Source: Transl Vis Sci Technol. 2021 Jul 27;10(8):24. doi: 10.1167/tvst.10.8.24 (PMC8322722; doi:10.1167/tvst.10.8.24)
Supplement: Supplement 2 [file tvst-10-8-24_s002.docx]

# Supplementary Material

| Age group (years) | Number of participants | ≥1 ONH successful (%) | Bilateral ONH successful (%) |
| --- | --- | --- | --- |
| All* | 46 | 39 (85%) | 35 (76%) |
| Under 1 | 2 | 2 (100%) | 2 (100%) |
| 1-1.99 | 4 | 3 (75%) | 3 (75%) |
| 2-2.99 | 5 | 3 (60%) | 3 (60%) |
| 3-3.99 | 12 | 9 (75%) | 6 (50%) |
| 4-4.99 | 3 | 3 (100%) | 3 (100%) |
| 5-5.99 | 5 | 4 (80%) | 4 (80%) |
| 6 and over | 15 | 15 (100%) | 14 (93%) |

**Supplementary Table S1: ONH image acquisition success rates by age group.**

*Key:* *This table excludes four children imaged under general anaesthesia, aged 2, 6, 6 and 13, of which one 6-year-old was unsuccessful due to pinpoint pupils and eye-rolling secondary to opiate administration. Percentages reported as whole numbers. ONH = optic nerve head.

| Background information | Number |
| --- | --- |
| Patients with logMAR chart VA* | 31† |
| logMAR chart VA: median | 0.10 |
| logMAR chart VA: range | -0.06 to 1.30‡ |
| logMAR chart VA: IQR | 0.02 to 0.20 |
| Patients with satisfactory fundoscopic examination§ | 40# |
| Patients with papilloedema detected on fundoscopic examination | 1 |
| Patients with optic atrophy detected on fundoscopic examination | 1 |

**Supplementary Table S2: Visual acuity and fundoscopic findings.**

*Key:* *Out of 43 patients with at least one analysable ONH image, 12 children were too young and/or unable to cooperate with logMAR chart VA testing: three cooperated with Keeler Acuity Cards (2.1, 2.1 and 6.5 cycles per degree), five demonstrated fixing and following while four could not cooperate. †Of the 31 patients with logMAR VAs, these were measured on the same day as handheld OCT in all but three exceptions: i) 39 days prior; ii) 1 day prior and iii) 2 days post-OCT. ‡Amblyopic eye. §Out of 43 patients with at least one analysable ONH image, three fundoscopic examinations were unsatisfactory (corneal scar: n=1; fleeting views only: n=2). #Median time in days between fundoscopy and handheld OCT was 0 days (IQR 0 – 63.5) – discrepancies were due to patients being newly referred or having shared care with other hospitals. IQR = interquartile range; ONH = optic nerve head; VA = visual acuity.
